# Inequity-aversion and relative kindness intention jointly determine the expenditure of effort in project teams

**DOI:** 10.1371/journal.pone.0176721

**Published:** 2017-05-01

**Authors:** Jiaojie Han, Amnon Rapoport, Rui Zhao

**Affiliations:** 1 Department of Finance, Zhongnan University of Economics and Law, Wuhan, Hubei, China; 2 School of Business Administration, University of California, Riverside, California, United States of America; 3 Faculty of Geosciences and Environmental Engineering, Southwest Jiaotong University, Chengdu, Sichuan, China; Middlesex University, UNITED KINGDOM

## Abstract

The literature on team cooperation has neglected the effects of *relative kindness intention* on cooperation, which we measure by comparing the kindness intentions of an agent to her group members to the kindness shown by other members to this same agent. We argue that the agent’s emotional reaction to material payoff inequity is not constant, but rather affected by her *relative kindness intention*. Then, we apply the model to team projects with multiple partners and investigate how *inequity-aversion* and *relative kindness intention* jointly influence team cooperation. We first consider the case of homogeneous agents, where their marginal productivity levels and technical capacities are the same, and then consider the case of heterogeneous agents, where their marginal productivity levels and technical capacities are not the same. Our results show that inequity-aversion has no effect on effort expenditure in the former case, but does affect it in the latter case. The consideration of *relative kindness intention* may impact the agents’ optimal cooperative effort expenditure when their technical capacities are different. In addition, it is beneficial for team cooperation, and might not only reduce the negative impact but also enhance the positive impact of inequity-aversion on the agents’ effort expenditures.

## 1. Introduction

### 1.1 Material payoff and relative kindness

Project teams requiring multiple agents from different disciplines have become the subject of extensive research [1–4]. A major issue in the project team literature is how fairness (or inequity-aversion) impacts cooperation [5–7]. Theoretical and experimental studies of interactive decision behavior have advanced the proposition that fairness is critical for cooperative behavior [8–11]. Most of the existing literature on teamwork has proposed two factors that affect the preference for fairness: material payoffs and kindness intentions [12–13]. By the former, the agents experience inequality if and only if their payoffs are not equal [14–15], whereas by the latter, the agents are less affected by the inequality of their material outcomes if the other agents act as fairly as possible [16]. Progressing beyond the economic models that focus separately on material outcomes and kindness intentions, several recent studies have considered the *integration* of material payoffs and expressions of kindness. For instance, Falk and Fischbacher [17] argued that the underlying intention of an action affects the evaluation of its kindness in addition to the material consequences.

In sharp departure from previous studies, we find that the literature on kindness intention has considered separately the kindness intention of an agent (hereafter called the *pivotal agent*) and the kindness acts of other agents in the group (e.g., [18]), ignoring the interaction between them. In reality, the pivotal agent usually considers her kindness to other team members as a reference point (or benchmark) to evaluate the degree of kindness that other team members show to her. In a theoretical and experimental study, Celen et al. [19] stated that “players make judgments about kindness through comparing the kindness of their own with their opponents.” These authors offer a novel definition of kindness as a relative concept. They argue that for player *j* to judge whether player *i* is kind to her, player *j* has to put herself in the position of player *i*. Like Celen et al. [19], we propose that an agent considers the *relative* kindness intention by comparing the kindness that other team members show to her to the kindness that she shows to them.

We also notice that fairness models considering material payoffs regard the marginal rate of substitution between the players’ payoffs to be constant [14]. However, as Cox et al. [20] argued convincingly, because the agents’ emotional states are affected by considerations of reciprocity and status, the marginal rate of substitution between one’s own material payoff and the material payoffs of others cannot be constant. Therefore, we argue that *relative kindness intention* is an important factor affecting the marginal rate of material payoffs that could further change the worker’s reaction to payoff inequity. We assume that if a focal agent perceives the other agents to be as kind as she is, then she experiences less inequity if her material outcome is smaller than the material outcome of her team members. Similarly, an agent feels less guilty when her payoff exceeds the payoffs of the other team members, if she has tried her best. Under these assumptions, we propose a utility function that allows the agents to compare their own kindness intentions and material payoffs with those of the other agents. From the model of Fehr and Schmidt [14], we identify two components of the agent’s preference for fairness, *relative material payoff* and *relative kindness intention*. As with the paper of Stanca [21], we modify the model proposed by Fehr and Schmidt [14], by incorporating the kindness intentions examined by Rabin [22]. The model implies the hypothesis that, given her kindness intention, the agent with a payoff smaller than the payoffs of others may experience less inequality if the other agents are kind to her, and more inequality if they show no kindness to her. This hypothesis is consistent with the findings of Nelson [12], who indicated that people are more willing to accept the inequality caused by nature than that instigated by human behavior. This is also consistent with Ku and Salmon [23], who found that the tolerance of individuals to inequity depends on the perceived fairness of the procedures that have led to the inequity. Moreover, if the agent feels that the other agents are as kind as she is, then her sense of inequality would mainly depend on her relative material payoff, and consequently the impact of the kindness intention on her sense of inequity would vanish.

### 1.2 Homogeneous and heterogeneous agents

In this paper, we consider team projects in which the project manager (hereafter called *principal*) cannot observe the individual efforts of the team members (hereafter called *agents*). We also assume that the principal cannot identify the agents’ skill differentials correctly and hence cannot compensate them contingent on their individual performance. Under these circumstances, the principal may only offer a group-based contract to the agents, which compensates them equally.

In the context of a project team under a group-based contract, the agents are usually deemed to be homogeneous (symmetric) and hence they are expected to expend the same level of effort to decrease the negative utility associated with inequality [3, 24–25]. However, homogeneous groups are a theoretical construct seldom observed in practice. In general, agents differ from one another in multiple characteristics, including physical strength, technical skills, intelligence, experience, and capacities. In recent years, several studies have demonstrated how equity affects cooperation in groups of heterogeneous agents. For instance, Reuben and Riedl [26–27] argued in two papers that if the groups include heterogeneous members, then it would be difficult to achieve cooperation through informal sanctions. Kölle [28] investigated how heterogeneity, both in capacity and valuation, affects cooperation in public goods games, and Kube et al. [10] studied how instruction is affected by the heterogeneity of the benefits of players from both their cooperation and institutional obligations. Other researchers have studied the impact of asymmetries on cooperation without considering the preference for fairness. For instance, Dubois and Vukima [29] investigated the principal’s contract design when agents are heterogeneous in their risk preferences and costs of effort, and Hartig et al. [30] analyzed how heterogeneous contributions affect the individual contributions of agents in a single-shot linear public game.

The above-mentioned studies focus on the impact of heterogeneity of group members on their cooperation mostly in the context of experiments on public good games, without discussing the effectiveness of the *relative kindness intention*. Although the public good game has been widely discussed in the literature on cooperative behavior, it may not account for the cooperation in project teams. Cooperation is considerably more complex in project teams with multiple partners than in public good games, and the agents in project teams are more likely to be heterogeneous. In this paper, we examine the effects of two major sources of heterogeneity, namely, *technical capacity* and *marginal productivity*, on the willingness to cooperate in multi-partner project teams. The agents’ technical capacities are determined by their work experience and professional qualifications, whereas their marginal productivities are determined by their personal characteristics and productivity levels. It is natural for the agents who work jointly on a project to have different technical capacities as well as different marginal productivities. Moreover, the marginal productivity of an agent in a given project may differ across different stages. For example, in a construction project, the agent assuming responsibility for the project’s design is expected to have a higher marginal productivity in the design stage than in the implementation stage. Similarly, agents with the same marginal productivity may have different technical capacities. For instance, a principal may hire two or more agents for the same kind of task whose productivity levels are equal but their technical capacities are not.

To sum up, we posit that the literature on team projects has neglected the effect of relative kindness intentions on cooperation, mostly restricting its discussion to the traditional public good game [10, 13]. Our paper examines the expenditure of effort in multi-partner project teams, where the agents may be heterogeneous in terms of their capacity level and marginal productivity, and are motivated to cooperate with one another in order to complete the project successfully. Instead of assuming that the preference for equity depends on the relative material payoffs, we consider the case where it depends on both the relative material payoff and relative kindness intention. The insights gained from this study may assist managers to better understand the cooperative behavior of group members in team projects as well as provide the foundations for mechanism design.

Through theoretical and numerical analysis, we find that the consideration of relative kindness intention is important in project teams where agents are heterogeneous in their technical capacities. With the consideration of relative kindness intention in heterogeneous conditions, *high-capacity* agents tend to expend higher levels of effort than when they are selfish or consider inequity only in terms of material payoff. Moreover, in contrast to the previous literature reporting that the impact of inequity-averse preferences on cooperation under group-based contract is negative (e.g., [10]), as well as the literature reporting that inequity-aversion promotes cooperation (e.g., [11,31]), we show that the effect of inequity-aversion and the consideration of relative kindness intention in project teams are beneficial for team cooperation when the agents have heterogeneous productivities and homogeneous capacities, or in team projects with high-effort marginal cost. However, they are harmful for cooperation in team projects with low-effort marginal costs. In addition, the consideration of relative kindness intention is beneficial for team cooperation by magnifying the positive effect of inequity-aversion and reducing its negative effect. Our theoretical results imply that it is beneficial to hire agents with different productivities but similar capacities to enhance team cooperation. The project manager may promote team cooperation through a training program that enhances the ability of the low-capacity agent or improves the information symmetry between the two agents.

The rest of this paper is organized as follows. Section 2 describes the model setting. Section 3 proposes equilibrium solutions for symmetric and asymmetric conditions and outlines managerial insights. The final section presents our concluding remarks.

## 2. The model

### 2.1 Basic assumptions

Consider a project team consisting of a principal and two agents with possibly different technical skills, indexed by, *j* ∈ {1,2}, *i ≠ j*. Assume that the principal and the two agents are risk-neutral. The agents work jointly on a project to produce some good (product) or perform some task that cannot be completed by either one of them singly; in order to complete the project, the agents ought to cooperate with each other. Let *b*_*i*_ ∈ [0,1] denote the effort that agent *i* expends on the project. Assume that the effort cost function for this agent is given by $C_{i}\left(b_{i}\right)=\frac{1}{2}cb_{i}^{2}$, where the cost parameter *c (c > 0)* is constant for both agents. Obviously, *C*_*i*_ (*b*_*i*_) is increasing in *b*_*i*_, strictly convex, and twice continuously differentiable; that is, ∂ *C*_*i*_ (*b*_*i*_) / *∂b*_*i*_ > 0 and $\partial C_{i}^{2}\left(b_{i}\right)/\partial b_{i}^{2}>0$.

Let *f* denote the production function of the project, which is determined by the agents’ individual efforts, and assume that it is increasing in both of its arguments and is continuously differentiable; that is, ∂*f* / ∂*b*_*i*_ ≥ 0, *∂f* / *∂b*_*j*_ ≥ 0, $\partial^{2}f/\partial b_{i}^{2}\leq 0$, $\partial^{2}f/\partial b_{j}^{2}\leq 0$, and *f*(0,0) = 0. Because the agents could be endowed with different technical skills, we assume that the project’s output when *b*_*i*_ ≠ 0 and *b*_*j*_ ≠ 0 exceeds the sum of the individual outputs; that is, *f*(*b*_*i*_, *b*_*j*_) > *f*(*b*_*i*_, 0) + *f*(0, *b*_*j*_). Therefore, there should be a collaborative output component in the productive function.

Kretschmer and Puranam [32] proposed a production model that satisfies our assumptions. On the basis of their production model, we specify the form of production function *f*(*b*_*i*_, *b*_*j*_) as follows:

$$f=\theta b_{i}+\left(1-\theta \right)b_{j}+\gamma b_{i}b_{j}+\xi ,$$ (1)

where the parameter *ξ* is a random variable with *E*(*ξ*) = 0 and *var*(*ξ*) = *σ*^2^ that represents the influence of the environmental factors not accounted for in our model. The nonnegative parameters *γ* and *θ* ∈ [0,1] represent the contribution of the collaboration efforts and the productivity of agent *i* on the project output, respectively. If *θ* = 0.5, then the efforts of the two agents are equally important to the project’s output.

When the project is completed, the outcome is scored as either success or failure with respective utilities *V* (*V* > 0) and 0. Here, *V* is the *project value*. Assume that the (exogenous) probability of success *P*(*f*) is positively related to the project’s output *f*, *P*(*f*) ∈ [0,1], and that the more the joint effort of the agents in the project, the more likely is the project’s success; that is, *P*(*b*_*i*_, *b*_*j*_) > *P*(*b*_*i*_ − Δ, *b*_*j*_) > *P*(*b*_*i*_ − Δ, *b*_*j*_ − Δ) and *P*(*b*_*i*_, *b*_*j*_) > *P*(*b*_*i*_, *b*_*j*_ − Δ) > *P*(*b*_*i*_ − Δ, *b*_*j*_ − Δ), where 1 ≥ *b*_*i*_, *b*_*j*_ ≥ Δ and 1 > Δ > 0. Without loss of generality, the probability function is assumed to be linear in the project’s output: *P*(*f*) = *τE*(*f*), where *τ* > 0. If neither of the two agents exerts any effort, then the project will terminate with probability 0. If each of them exerts 1 unit of effort, then the project will succeed with probability 1. Since *P*(*f*) ∈ [0,1] and *P*(*f*(1,1)) = 1, the value of parameter *τ* should be equal to $\frac{1}{1+\gamma }$.

Like the team setups examined in previous studies [33–36], the principal in our model cannot monitor the individual effort expended by either agent, but the agents can observe each other’s contributions to the project’s output. The principal can only find out whether the project is successful. Thus, in this paper, the principal is assumed to offer an incentive contract based on the (binary) group output. If the project succeeds, then each agent will receive individual reward *ηV* offered by the principal, where the parameter *η* denotes the *proportion* of *V* that the principal shares with the two agents, and, otherwise, the agents receive no reward. Since there are two agents in our model, *η* should satisfy *η* ∈ (0,0.5]. From the above assumptions, in case the project output is *f*, an agent’s expected material payoff will be equal to *ηVP*(*f*). In addition to the individual rewards, we assume that the agents receive a regular salary *w* > 0, which is not contingent on the output of the project. Therefore, the expected monetary payoff of each agent is given by

Wage=w+ηVP(f). (2)

Let *π*_*i*_(*b*_*i*_, *b*_*j*_) denote the payoff of agent *i* when he expends *b*_*i*_ units of effort and agent *j* expends *b*_*j*_ units of effort. This payoff is equal to the monetary payoff that the principal offers to agent *i* minus her effort cost:

$$\pi_{i}\left(b_{i},b_{j}\right)=Wage-C\left(b_{i}\right).$$ (3)

In Section 2.2, we propose a utility function that includes a term for the payoff of the agent as well as terms for the kindness intentions that he confers on others and that the other agents confer on him.

### 2.2 Inequity-aversion model with relative kindness intentions

Let *ϕ*_*i*_ represent the kindness shown by agent *i* to agent *j*, and $\tilde{\phi_{j}}$ represent agent *i’*s beliefs about the kindness that agent *j* confers on agent *i*. We assume that agent *i*’s equitable feelings depend on both his kindness to agent *j* and agent *j*’s kindness to him. Then, agent *i*’s *relative kindness intention* to agent *j* is defined as

$$\varphi_{i,j}=\phi_{i}-\tilde{\phi_{j}}.$$ (4)

Following Rabin [22], *ϕ*_*i*_ and $\tilde{\phi_{j}}$ are defined as follows:

$$\phi_{i}=\frac{\pi_{j}\left(b_{i},b_{j}^{\prime }\right)-\pi_{j}^{e}\left(b_{j}^{\prime }\right)}{\pi_{j}^{h}\left(b_{j}^{\prime }\right)-\pi_{j}^{min}\left(b_{j}^{\prime }\right)}\text{ and }\tilde{\phi_{j}}=\frac{\pi_{i}\left(b_{i}^{\prime \prime },b_{j}\right)-\pi_{i}^{e}\left(b_{i}^{\prime \prime }\right)}{\pi_{i}^{h}\left(b_{i}^{\prime \prime }\right)-\pi_{i}^{min}\left(b_{i}^{\prime \prime }\right)}\;,$$ (5)

where *b*_*i*_ (or *b*_*j*_) denotes the effort level chosen by agent *i* (or agent *j*), $b_{j}^{\prime }$ (or $b_{i}^{\prime }$) denotes agent *i*’s (or agent *j*’s) beliefs about the effort level agent *j* (or agent *i*) will choose to expend, and $b_{i}^{\prime \prime }$ (or $b_{j}^{\prime \prime }$) denotes agent *i*’s (or agent *j*’s) belief about what agent *j* (or agent *i*) believes about agent *i*’s (or agent *j*’s) effort choice. $\pi_{j}^{h}\left(b_{j}^{\prime }\right)$ and $\pi_{j}^{min}\left(b_{j}^{\prime }\right)$ represent agent *j*’s highest and lowest payoffs, respectively, when he expends $b_{j}^{\prime }$ units of effort. $\pi_{i}^{h}\left(b_{i}^{\prime \prime }\right)$ and $\pi_{i}^{min}\left(b_{i}^{\prime \prime }\right)$ denote agent *i*’s highest and lowest payoffs, respectively, when he expends $b_{i}^{\prime \prime }$ units of effort. $\pi_{j}^{e}\left(b_{j}^{\prime }\right)$ and $\pi_{i}^{e}\left(b_{i}^{\prime \prime }\right)$ are the equitable payoffs of agent *i* and agent *j*, respectively. From Eq (3), $\pi_{j}^{h}$, $\pi_{j}^{min}$, and $\pi_{j}^{e}$ can be defined as follows:

1. $\pi_{j}^{h}\left(b_{j}^{\prime }\right)={\text{max}}_{b_{i}}\pi_{j}\left(b_{i},b_{j}^{\prime }\right)=w+\eta VP\left(f\left(b_{i}{}^{max},b_{j}^{\prime }\right)\right)-\frac{1}{2}cb_{j}^{\prime }{}^{2};$
2. $\pi_{j}^{min}\left(b_{j}^{\prime }\right)={\text{min}}_{b_{i}}\pi_{j}\left(b_{i},b_{j}^{\prime }\right)=w+\eta VP\left(f\left(0,b_{j}^{\prime }\right)\right)-\frac{1}{2}cb_{j}^{\prime }{}^{2};$
3. $\pi_{j}^{e}\left(b_{j}^{\prime }\right)=\frac{1}{2}\left(\pi_{j}^{h}\left(b_{j}^{\prime }\right)+\pi_{j}^{l}\left(b_{j}^{\prime }\right)\right).$

$\pi_{j}^{l}\left(b_{j}^{\prime }\right)$ is the minimum of the payoffs agent *j* can earn, given $b_{j}^{\prime }$. Since agent *j*’s payoff increases in agent *i*’s effort, the latter payoff depends on agent *i*’s worst response to agent *i*’s beliefs about agent *j*’s effort choice. Therefore, $\pi_{j}^{l}\left(b_{j}^{\prime }\right)=\pi_{j}^{min}\left(b_{j}^{\prime }\right)$. By incorporating these expressions into Eq (4), we obtain

$$\phi_{i}=\frac{b_{i}}{b_{i}{}^{max}}-\frac{1}{2}.$$ (6)

The kindness shown by agent *j* to agent *i* is obtained similarly:

$$\tilde{\phi_{j}}=\frac{b_{j}}{b_{j}{}^{max}}-\frac{1}{2},$$ (7)

where $b_{i}^{max}\in (0,1]$ and $b_{j}^{max}\in (0,1]$ stand for agent *i*′*s* and agent *j*’s technical capacity, respectively; these represent the maximal levels of effort that they can expend on the project. Furthermore, Eqs (6) and (7) jointly show that the agents’ kindness intentions to others are determined by their expended efforts and technical capacity.

By incorporating Eqs (6) and (7) into Eq (4), agent *i*’s relative kindness intention to agent *j* becomes equivalent to

$$\varphi_{i,j}=\frac{b_{i}}{b_{i}{}^{max}}-\frac{b_{j}}{b_{j}{}^{max}}.$$ (8)

The constraints on *b*_*i*_ and *b*_*j*_ imply that the value of *φ*_*i*,*j*_ is contained in the range [−1,1]. *φ*_*i*,*j*_ < 0 indicates that agent *i* is mostly unkind to agent *j* although agent *j* is mostly kind to agent *i*, whereas *φ*_*i*,*j*_ = 1 means that agent *i* is mostly kind to agent *j* although agent *j* is mostly unkind to agent *i*. *φ*_*i*,*j*_ > 0 means that agent *i* is more kind than agent *j*, and *φ*_*i*,*j*_ < 0 means that agent *j* is more kind than agent *i*. If *φ*_*i*,*j*_ = 0, then agent *i* is as kind as agent *j*. In practice, each agent can only estimate the other agent’s relative kindness intention, but cannot measure it precisely. Therefore, in order to simplify the mathematical analysis, we assume that *φ*_*i*,*j*_ is a fuzzy variable defined by

$$\varphi_{i,j}=\{\begin{array}{ll} 1 & if\;\phi_{i}-\tilde{\phi_{j}}=1\\ \delta_{i} & if\;1>\phi_{i}-\tilde{\phi_{j}}>0\\ 0 & if\;\phi_{i}=\tilde{\phi_{j}}\\ -\delta_{i} & if\;0>\phi_{i}-\tilde{\phi_{j}}>-1\\ -1 & if\;\phi_{i}-\tilde{\phi_{j}}=-1 \end{array},$$ (9)

where *δ*_*i*_ ∈ (0,1) indicates agent *i*’s feeling about unkindness when he is kinder than agent *j*, and −*δ*_*i*_ ∈ (−1,0) indicates his feeling about unkindness when he is less kind than agent *j*. For the sake of simplicity, we assume that agent *i*’s feelings about unkindness is a constant equal to *δ* if *φ*_*i*,*j*_ ∈ (0,1), and to −*δ* if *φ*_*i*,*j*_ ∈ (−1,0).

Let Δ_*i*,*j*_ denote the *payoff difference* between agents *i* and *j*:

$$\Delta_{i,j}=\pi_{i}\left(b_{i}\right)-\pi_{j}\left(b_{j}\right).$$ (10)

If Δ_*i*,*j*_ < 0 (*π*_*i*_ < *π*_*j*_), then agent *i* experiences less inequality when agent *j* is kinder to agent *i* than agent *i* is to agent *j* (*φ*_*i*,*j*_ < 0), and that agent *i* experiences more inequality when this situation is reversed (*φ*_*i*,*j*_ > 0). Similarly, if Δ_*i*,*j*_ > 0, then agent *i* experiences more inequality when *φ*_*i*,*j*_ < 0 and less inequality when *φ*_*i*,*j*_ > 0. If we incorporate the relative kindness intention into the inequity function of Fehr and Schmidt [14], then agent *i*’s utility function can be expressed as follows:

$$U_{i}=\pi_{i}-\alpha_{i}\frac{1}{n-1}\sum_{j\neq i}\text{max}\left\{\left(1+\varphi_{i,j}\right)\Delta_{j,i},0\right\}-\beta_{i}\frac{1}{n-1}\sum_{j\neq i}\text{max}\left\{\left(1-\varphi_{i,j}\right)\Delta_{i,j},0\right\}.$$ (11)

Here, the parameter *α*_*i*_ measures how much agent *i* dislikes disadvantageous inequity, that is, by how much the other agent’s payoff is higher than agent *i*’s payoff. *β*_*i*_ represents how much agent *i* dislikes his payoff being higher than the other agents’ payoffs (advantageous inequity). Because economic agents usually experience more inequality when their payoff is smaller than the payoffs of others, like Fehr and Schmidt [14], we assume that 0 < *β*_*i*_ ≤ 1 ≤ *α*_*i*_. For the sake of simplicity, we assume that the agents’ feelings about relative material payoff and relative kindness intention are constant across the agents; that is, *α*_*i*_ = *α*_*j*_ = *α*, *β*_*i*_ = *β*_*j*_ = *β* and *δ*_*i*_ = *δ*_*j*_ = *δ*.

Similar to our notion of relative kindness intention, Celen et al. [19] considered the notion of kindness as a relative rather than absolute concept. They proposed a model incorporating the notion of blame, assuming that agent *i*’s utility *u*_*i*_ is determined by the sum of his material payoff and a proportion of the other player’s material payoff. Our model is different from their model in two major aspects. First, in their model, the blame that one player confers on other players is determined by the other players’ actions across all stages of the game. In contrast, in our model, we focus on whether the agents expend equitable efforts. In the spirit of Rabin [22], we assume that an agent assesses the kindness of others by comparing their actual expenditures to what he expects them to expend. Second, we propose a model that allows for *both* the relative material payoff and relative kindness intention, whereas they do not consider the relative material payoff at all. In reality, when people evaluate fairness, material payoff is one of the key factors that should not be ignored [8–11]. Therefore, the contribution of our model is in incorporating relative kindness intention into the notion of inequity-aversion and assuming that it affects the agents’ feelings about the inequity caused by the difference between the two material payoffs.

Using the solution concept of the fairness equilibrium proposed by Rabin [22], we formulate multi-partner cooperation in project teams, where the players maximize their utilities, as follows:

$$\{\begin{array}{l} U_{i}=\pi_{i}-\alpha max\left\{\left(1+\varphi_{i,j}\right)\Delta_{j,i},0\right\}-\beta max\left\{\left(1-\varphi_{i,j}\right)\Delta_{i,j},0\right\}\\ U_{j}=\pi_{j}-\alpha max\left\{\left(1+\varphi_{j,i}\right)\Delta_{i,j},0\right\}-\beta max\left\{\left(1-\varphi_{j,i}\right)\Delta_{j,i},0\right\}\\ U_{i}\geq 0,\;U_{j}\geq 0,\\ b_{i}^{*}=\text{arg}maxU_{i}\;,b_{j}^{*}=\text{arg}maxU_{j}\\ b_{i}^{*}=b_{i}^{\prime }=b_{i}^{\prime \prime },b_{j}^{*}=b_{j}^{\prime }=b_{j}^{\prime }{}^{\prime },\;i,j\in \left\{1,2\right\},\;i\neq j \end{array}$$ (12)

## 3. Main results

In this section, we present some theorems about the effects of inequity-aversion and the consideration of relative kindness intention on the agents' effort expenditures in both symmetric and asymmetric conditions. Note that “inequity-aversion” only represents the agents’ feelings about the inequity-aversion caused by the relative material payoff. In addition, since we focus on how inequity-aversion and the relative kindness intention affect the agents’ expenditure levels, we ignore the incentive of the principal, assuming that the profit share parameter *η* is fixed. The proofs are relegated to the appendix.

### 3.1. Symmetric case analysis

Suppose that the agents are identical, that is *θ* = 0.5 and $b_{i}^{max}=b_{j}^{max}=1$. By maximizing the agents’ utilities in Eq (12), we obtain the following proposition:

**PROPOSITION 1.** *In the symmetric condition*, *inequity-aversion and relative kindness intention do not affect the agents’ effort expenditure*. The agent’s optimal effort expenditure is (b*|_*sym*_, b*|_*sym*_ *)*, *where*

$$b^{*}{|}_{sym}=\{\begin{array}{ll} \frac{\eta V\tau }{2\left(c-\eta V\tau \gamma \right)}, & c>\eta V\tau \gamma \\ 1, & c\leq \eta V\tau \gamma \end{array}.$$

Proposition 1 implies that although the agents consider the inequity induced by the relative material payoff and relative kindness intentions, in the symmetric condition no agent is willing to expend a level of effort different from b*|_*sym*_. The same result obtains when the two agents are assumed to be selfish and only consider the inequity induced by the relative material payoff.

### 3.2. Asymmetric case analysis

In this section, we separately investigate two kinds of heterogeneity, one due to technical capacity, and the other to the efforts’ marginal productivity. In our model, technical capacity is represented by the agents’ maximal effort level that they can expend, that is, $b_{j}^{max}$. The efforts’ marginal productivity is represented by the marginal contribution of the agents’ efforts that they expend on the project output, that is, *θ*. We also consider two asymmetric scenarios. In Scenario 1, the agents’ technical capacities are the same but their marginal productivities are different, and in Scenario 2, the agents’ technical capacities are different and their marginal productivity levels are either different or identical.

#### 3.2.1. Scenario 1: Heterogeneous marginal productivity

In this section, we extend the analysis to heterogeneous agents, assuming that their efforts’ marginal contributions to the team output are different; that is, *θ* ≠ 0.5. With no loss of generality, we assume that agent *i*’s marginal contribution exceeds the marginal contribution of agent *j* (*θ* > 0.5). Hereafter, we call agent *i* the *high-productive* agent and agent *j* the *low-productive* agent. We can show that if the agents are selfish, then the optimal effort expenditures of agents *i* and *j* are, respectively,

$$b_{i}{}^{*}=\{\begin{array}{cc} \frac{\eta V\tau \left[\eta V\tau \gamma \left(1-\theta \right)+c\theta \right]}{c^{2}-\eta^{2}V^{2}\tau^{2}\gamma^{2}} & c>\eta V\tau \gamma \\ 1 & c\leq \;\eta V\tau \gamma \end{array},$$

$$b_{j}{}^{*}=\{\begin{array}{cc} \frac{\eta V\tau \left[\eta V\tau \gamma \theta +c\left(1-\theta \right)\right]}{c^{2}-\eta^{2}V^{2}\tau^{2}\gamma^{2}} & c>\eta V\tau \gamma \\ 1 & c\leq \eta V\tau \gamma \end{array}.$$ (13)

We define $b_{i}{}^{*}{|}_{S1}=\frac{\eta V\tau \left[\eta V\tau \gamma \left(1-\theta \right)+c\theta \right]}{c^{2}-\eta^{2}V^{2}\tau^{2}\gamma^{2}}$ and $b_{j}{}^{*}{|}_{S1}=\frac{\eta V\tau \left[\eta V\tau \gamma \theta +c\left(1-\theta \right)\right]}{c^{2}-\eta^{2}V^{2}\tau^{2}\gamma^{2}}$, where *S* stands for selfishness. A comparison of $b_{i}{}^{*}{|}_{S1}$ and $b_{j}{}^{*}{|}_{S1}$ shows that $b_{i}{}^{*}{|}_{S1}\;>\;b_{j}{}^{*}{|}_{S1}$ for *c > ηVτγ* and *θ* ≠ 0.5. This implies that in the completely selfish case, the *high-productive* agent would expend more effort than the *low-productive* agent. In the rest of this section, we investigate how inequity-aversion affects the agents’ optimal effort provisions.

**PROPOSITION 2.** *In condition θ* ≠ 0.5 *where* $b_{i}^{max}=b_{j}^{max}=1$, *if the agents are inequity-averse and consider the relative kindness intention*, then (b*|_asym1_, b*|_asym1_ *) is an equilibrium*, *where*

$$b^{*}{|}_{asym1}=\{\begin{array}{ll} \frac{\eta V\tau }{c-2\eta V\tau \gamma }, & c>2\eta V\tau \gamma \\ 1, & c\leq 2\eta V\tau \gamma \end{array}.$$

Proposition 2 implies that with inequity-aversion the agents would expend the same level of effort. This situation is different from the selfish condition, but it is the same as the condition where the agents only consider the relative material payoff. From a comparison of the agents’ effort expenditures, if the agents are selfish or have inequity-averse preferences, then we obtain the following corollary.

**Corollary 1.** *Assume that θ* ≠ 0.5 *and $b_{i}^{max}=b_{j}^{max}=1$. A comparison of the completely selfish agent with the inequity-averse agent shows that the agent expends higher efforts in the latter case. In this case, the consideration of relative kindness intention does not affect the agents’ optimal effort expenditures.*

From Corollary 1, in case the two agents are identical in their technical capacities, the inequity-averse preference induces the agents to expend the same level of cooperative effort regardless of their consideration of relative kindness intention. In addition, the result in Corollary 1 is different from Kube et al. [10], who reported that when offering a symmetric contract (group-based contract) to a team with asymmetric players, inequity-averse preferences could hamper team cooperation. Their result is predicated on the assumption that the agents are heterogeneous in their marginal benefits in the context of the public good game, which is quite different from ours. In our study, we find that when the agents are heterogeneous only in their productivity, inequity-aversion is *beneficial* for team cooperation. The intuition is that when the productivity levels are different but the capacities are the same, the high-productive agent will expend more effort than the low-productive agent in order to enhance his material payoff. The low-productive agent will incur a psychological cost when his effort expenditure is smaller than the expenditure level of the high-productive agent. Besides, a group-based contract may cause the high-productive agent to feel treated inequitably when he is rewarded at the same level as the low-productive agent. Therefore, in order to eliminate the negative emotions associated with inequity-aversion, the low-productive agent would attempt to match the effort of the high-productive agent. Corollary 1 suggests that the conclusions drawn from public good games with heterogeneous players should not be generalized to project teams, and that different assumptions about heterogeneity may yield different results. Ours is not the only study to report that inequity-aversion may have positive effects on cooperation and team performance. For instance, Qin et al. [31] show experimentally that concerns of fairness lead to greater supply-chain profits and a more balanced supply-chain profit distribution.

Other characteristics of the project, besides material payoffs and kindness intentions, may also affect the agents' effort expenditures, as summarized in Corollary 2.

**Corollary 2.** *In condition* ≠ 0.5, *where $b_{i}^{max}=b_{j}^{max}=1$, with inequity-aversion, the agents’ optimal effort expenditures are positively correlated with the marginal effort profits (ηVτγ) and negatively correlated with the marginal cost (c)*.

As with the selfish and symmetric conditions discussed above, the agents’ effort expenditures in this case are also increasing with the effort’s marginal profit and decreasing with the marginal cost. Corollary 2 indicates that even with social preferences such as inequity-aversion, monetary benefit is still a key factor in determining the agents’ effort expenditure.

#### 3.2.2. Scenario 2: Heterogeneous technical capacities

In this section, we extend the model discussed in Section 3.2.1 to the case of agents with different capacities. Suppose that one of the two agents, say agent ***i***, has high capacity (called *high-capacity agent*), and the other, say agent ***j***, has low capacity (called *low-capacity* agent); that is, $0<b_{j}^{max}<b_{i}^{max}=1$.

Generally, the higher the agent’s capacity, the more important is his contribution to the project. Therefore, in this part, we assume that the marginal productivity level of agent *i* is higher than or equal to that of agent *j*; that is *θ* ≥ 0.5. In case *θ* > 0.5, if $b_{j}^{max}>b^{*}{|}_{asym1}$, then the results are the same as in Section 3.2.1. Therefore, in this section, we assume that $b_{j}^{max}<b^{*}{|}_{asym1}$. Now, the optimal effort is that agent *j* expends $b_{j}^{max}$, and we obtain the following proposition:

**PROPOSITION 3.** *Let $c_{4}=\frac{\eta V\tau \left(\theta +\gamma b_{j}^{max}\right)}{\left[1+\alpha \left(1-\delta \right)\right]b_{j}^{max}}$, and $c_{5}=\frac{\eta V\tau \left(\theta +\gamma b_{j}^{max}\right)}{\left[1-\beta \left(1+\delta \right)\right]b_{j}^{max}}$. With inequity-aversion and the consideration of relative kindness intentions, if, θ* ≠ 0.5, $0<b_{j}^{max}<b_{i}^{max}=1$, *and* $b_{j}^{max}<b^{*}{|}_{asym1}$, *then* $\left(b_{i}^{*}{|}_{asym2},b_{j}^{max}\right)$ *is an equilibrium*, *where*

$$b_{i}^{*}{|}_{asym2}=\{\begin{array}{ll} \frac{\eta V\tau \left(\theta +\gamma b_{j}^{max}\right)}{c\left[1+\alpha \left(1-\delta \right)\right]}, & c<c_{4}\\ b_{j}^{max}, & c_{4}\leq c\leq c_{5}\\ \frac{\eta V\tau \left(\theta +\gamma b_{j}^{max}\right)}{c\left[1-\beta \left(1+\delta \right)\right]}, & c>c_{5} \end{array}.$$

Similar to Proposition 3, we obtain Proposition 4 as follows:

**PROPOSITION 4.** *Let $c_{6}=\frac{\eta V\tau \left(0.5+\gamma b_{j}^{max}\right)}{\left[1+\alpha \left(1-\delta \right)\right]b_{j}^{max}}$, and* $c_{7}=\frac{\eta V\tau \left(0.5+\gamma b_{j}^{max}\right)}{\left[1+\beta \left(1-\delta \right)\right]b_{j}^{max}}$. *With inequity-aversion and the consideration of relative kindness intentions*, *in case θ* = 0.5, $0<b_{j}^{max}<b_{i}^{max}=1$, *and* $b_{j}^{max}<b^{*}{|}_{sym}$, *then* $\left(b_{i}^{*}{|}_{asym3},b_{j}^{max}\right)$ *is an equilibrium, where*

$$b_{i}^{*}{|}_{asym3}=\{\begin{array}{ll} \frac{\eta V\tau \left(0.5+\gamma b_{j}^{max}\right)}{c\left[1+\alpha \left(1-\delta \right)\right]}, & c<c_{6}\\ b_{j}^{max}, & c_{6}\leq c\leq c_{7}\\ \frac{\eta V\tau \left(0.5+\gamma b_{j}^{max}\right)}{c\left[1-\beta \left(1+\delta \right)\right]}, & c>c_{7} \end{array}.$$

As the results for the two cases of *θ* ≠ 0.5 and *θ* = 0.5 are similar, in the following part we mainly discuss the former case *θ* ≠ 0.5. As shown in the appendix, we find that if *c* < *c*_4_, then $b_{i}^{*}{|}_{asym2}>b_{j}^{max}$, and if *c* > *c*_5_, then $b_{i}^{*}{|}_{asym2}<b_{j}^{max}$. Therefore, keeping the other parameters fixed, an increase in the effort’s marginal cost *c* decreases agent *i*’s willingness to expend more effort than agent *j* ($\partial b_{i}^{*}{|}_{asym2}/\partial c\leq 0$). Since efforts are costly, it is obvious that the high-capacity agent’s best effort expenditure is determined by the marginal cost *c*.

As noted by Ku and Salmon [22], when an improvement of social efficiency (as well as group efficiency) is mainly in favor of the rich, both the advantaged (rich) and disadvantaged (poor) agents do not expend the effort levels that would maximize their own income and social welfare. In our case, when the effort expenditure of the high-capacity agent exceeds the effort of the low-capacity agent, that is, $b_{i}^{*}{|}_{asym2}>b_{j}^{max}$, then the low-capacity agent benefits from the high-capacity agent’s effort expenditure, and this increases the difference of payoff between the two agents. This could explain why agent *i* does not expend the effort level that would maximize his payoff and the team output across all situations.

From Proposition 3, we obtain Corollary 3 as follows:

**Corollary 3.** *The consideration of relative kindness intentions is positively correlated with agent i’s optimal effort expenditure except when c*_4_ ≤ *c* ≤ *c*_5_.

We obtain similar results from Proposition 4. From Corollary 3, in this case the agents’ aversion to relative unkindness enhances the positive impact of inequity-aversion on project team cooperation. This might be the case because with the difference in capacities, the consideration of relative kindness intentions could weaken the aversion of the high-capacity agent to the low-capacity agent, and could further increase the agents’ effort expenditure and facilitate team cooperation. This result is consistent with previous research findings that people dislike the inequity caused by subjective reasons such as free-riding, but are more tolerant to the inequity caused by objective reasons such as the difference in their capacity [12, 22].

Furthermore, Proposition 3 and Corollary 3 imply that in Scenario 2 both inequity-aversion and the consideration of relative kindness intention can influence the agents’ best individual effort expenditures. This result is different from Scenario 1 in Section 3.2.1, where the consideration of relative kindness intention could not impact the agents’ best individual effort provision as well as the homogeneous condition in Section 2. From these results, the consideration of relative kindness intention can impact the agents’ best individual effort expenditures if and only if the agents’ capacities are heterogeneous.

After comparing the optimal effort choices of the two agents when they are inequity-averse, or, alternatively, when they are selfish, Corollary 4 below shows how the preferences for inequity-averse and relative kindness intention affect the agents’ effort choices.

**Corollary 4.** *Let $c_{8}=\frac{1}{b_{j}^{max}}\eta V\tau \left(\theta +\gamma b_{j}^{max}\right)$. Consider the selfish condition when θ* ≠ 0.5, $0<b_{j}^{max}<b_{i}^{max}=1$, *and* $b_{j}^{max}<b^{*}{|}_{asym1}$. *If c < c*_8_, *then inequity-aversion has a negative effect on agent i’s optimal effort expenditure*, *and if c < c*_4_, *then the consideration of relative kindness intention can diminish the negative impact*. *If c > c*_8_, *then inequity-aversion has a positive effect on agent i’s optimal effort expenditure*, *and if c > c*_5_ *then the consideration of relative kindness intention can enhance the positive impact*. *if c = c*_8_, *then inequity-aversion and the consideration of relative kindness intention have no effect on agent i’s optimal effort expenditure*.

Proposition 3, Corollary 3, and Corollary 4 jointly suggest that the consideration of relative kindness intentions is beneficial for project team cooperation in capacity heterogeneous conditions. Moreover, Proposition 3 and Corollary 4 imply that the positive effect of inequity-aversion and the consideration of relative kindness intention on the agents’ effort expenditure and team cooperation increase with the marginal cost *c*. Therefore, in a project with difficult and complex tasks, if the agents are heterogeneous in their capacity, then inequity-aversion and the consideration of relative kindness intention are beneficial for team cooperation and project output. Otherwise, in a project with relatively easy tasks, selfishness is more beneficial. This result may be due to the difficulty of increasing the profit with the marginal cost, and the probability of free riding by selfish agents increasing with the marginal cost as well. With inequity-averse preferences, agents attempt to expend the same effort level. Therefore, in a project where the marginal cost is low, a selfish agent would like to expend more efforts in order to maximize his payoff, whereas a fairness-minded agent would tend to expend the same effort level as the other agent. Otherwise, in a project where the marginal cost is high, a selfish agent could derive more benefit from free-riding. Therefore, in order to maximize his payoff, he would expend less effort than the other agent, whereas a fairness-minded agent would expend the same effort level as the other agent in order to reduce the negative effect caused by inequity in payoff. Thus, with a high marginal cost, inequity-aversion may reduce the agents’ willingness of free-riding and enhance the benefit to team cooperation and project output.

The following corollary describes the effects of other characteristics of both the project and agents, in addition to *c* and *δ*, on the agents’ effort expenditures.

**Corollary 5.** *With inequity-aversion and the consideration of relative kindness intention, the effort’s marginal profit (η, V), the potential production of cooperation between agents (γ), agent i’s productiveness (θ), and agent j’s capacity ($b_{j}^{max}$) are all positively correlated with agent i’s optimal effort expenditure.*

From Proposition 3 and Corollary 4, we find that when *c* < *c*_4_, the best individual effort expenditure with inequity-aversion and the consideration of relative kindness intention is lower than in the selfish condition; that is, $b_{i}^{*}-b_{i}^{*}{}^{S}<0$. Therefore, increasing *η*, *V*, *γ*, and *θ* may reduce the negative effect of inequity-averse preferences on team cooperation. When *c* > *c*_5_, inequity-aversion and the consideration of relative kindness intention are beneficial for the agents’ best individual effort expenditure; that is, $b_{i}^{*}-b_{i}^{*}{}^{S}>0$. Therefore, when *c* > *c*_5_, raising *η*, *V*, *γ*, and *θ* enhances the positive effect of inequity-aversion and the consideration of relative kindness intention on team cooperation. Furthermore, improvement of the low-capacity agent’s ability ($b_{j}^{max}$) may enhance team cooperation. A practical implication of this is that a project manager may promote team cooperation through a training program that enhances the ability of the low-capacity agent or improves the information symmetry between the two agents.

### 3.3. Summary and discussion

The summary of our results is presented in Tables 1–3. In Tables 1 and 3, the symbol “⊗” represents no effect on the agents’ best individual effort expenditure, the symbol “+” represents a positive effect, and the symbol “−” represents a negative effect.

**Table 1:** The impact of inequity-aversion and consideration of relative kindness intention (RKI) on agents’ best individual effort compared with the selfish condition.

Conditions	Inequity-Aversion	RKI (*δ*)
*θ* = 0.5, $b_{i}^{max}=b_{j}^{max}=1$	⊗	⊗
*θ* ≠ 0.5, $b_{i}^{max}=b_{j}^{max}=1$	+	⊗
*θ* ≠ 0.5, $b_{i}^{max}\neq b_{j}^{max}$, $b_{j}^{max}<b^{*}{|}_{asym1}$	In case *c* < *c*_8_, − In case *c* > *c*_8_, + In case *c* = *c*_8_, ⊗	In case *c* < *c*_4_ and *c* > *c*_5_, + In case *c*_4_ ≤ *c* ≤ *c*_5_, ⊗
*θ* = 0.5, $b_{i}^{max}\neq b_{j}^{max}$, $b_{j}^{max}<b^{*}{|}_{sym}$	In case *c* < *c*_9_, − In case *c* > *c*_9_, + In case *c* = *c*_9_, ⊗	In case *c* < *c*_6_ and *c* > *c*_7_, + In case *c*_6_ ≤ *c* ≤ *c*_7_, ⊗

*Note*: $c_{9}=\frac{1}{b_{j}^{max}}\eta V\tau \left(\frac{1}{2}+\gamma b_{j}^{max}\right)$.

**Table 2:** Best individual effort expenditures with inequity-aversion and consideration of relative kindness intention.

Conditions	Best individual effort expenditure
***θ* = 0.5**, $b_{i}^{max}=b_{j}^{max}=1$	(b*|_*sym*_, b*|_*sym*_), where $b^{*}{|}_{sym}=\{\begin{array}{ll} \frac{\eta V\tau }{2\left(c-\eta V\tau \gamma \right)}, & c>\eta V\tau \gamma \\ 1, & c\leq \eta V\tau \gamma \end{array}$.
***θ* ≠ 0.5**, $b_{i}^{max}=b_{j}^{max}=1$	(b*|_*asym*1_, b*|_*asym*1_), where $b^{*}{|}_{asym1}=\{\begin{array}{ll} \frac{\eta V\tau }{c-2\eta V\tau \gamma }, & c>2\eta V\tau \gamma \\ 1, & c\leq 2\eta V\tau \gamma \end{array}$.
***θ* ≠ 0.5**, $b_{i}^{max}\neq b_{j}^{max}$, $b_{j}^{max}<b^{*}{|}_{asym1}$	$\left(b_{i}^{*},b_{j}^{max}\right)$, where $b_{i}^{*}{|}_{asym2}=\{\begin{array}{ll} \frac{\eta V\tau \left(\theta +\gamma b_{j}^{max}\right)}{c\left[1+\alpha \left(1-\delta \right)\right]}, & c<c_{4}\\ b_{j}^{max}, & c_{4}\leq c\leq c_{5}\\ \frac{\eta V\tau \left(\theta +\gamma b_{j}^{max}\right)}{c\left[1-\beta \left(1+\delta \right)\right]}, & c>c_{5} \end{array}$.
***θ* = 0.5**, $b_{i}^{max}\neq b_{j}^{max}$, $b_{j}^{max}<b^{*}{|}_{sym}$	$\left(b_{i}^{*},b_{j}^{max}\right)$, where $b_{i}^{*}{|}_{asym3}=\{\begin{array}{ll} \frac{\eta V\tau \left(0.5+\gamma b_{j}^{max}\right)}{c\left[1+\alpha \left(1-\delta \right)\right]}, & c<c_{6}\\ b_{j}^{max}, & c_{6}\leq c\leq c_{7}\\ \frac{\eta V\tau \left(0.5+\gamma b_{j}^{max}\right)}{c\left[1-\beta \left(1+\delta \right)\right]}, & c>c_{7} \end{array}$.

**Table 3:** The impact of the model’s parameters on the agents’ best individual effort.

Conditions	*ηVτγ*	*c*	*θ*	*α*	*β*	$b_{j}^{max}$
***θ* = 0.5**, $b_{i}^{max}=b_{j}^{max}=1$	+	−	⊗	⊗	⊗	⊗
***θ* ≠ 0.5**, $b_{i}^{max}=b_{j}^{max}=1$	+	−	⊗	⊗	⊗	⊗
***θ* ≠ 0.5**, $b_{i}^{max}\neq b_{j}^{max}$, $\;b_{j}^{max}<b^{*}{|}_{asym1}$	+	−	+	−	+	+
***θ* = 0.5**, $b_{i}^{max}\neq b_{j}^{max}$, $\;b_{j}^{max}<b^{*}{|}_{sym}$	+	−	⊗	−	+	+

Table 1 shows that, compared to the selfish condition, the impact of inequity-aversion and consideration of the relative kindness intention is quite different across the different cases. This finding implies that inequity-aversion does not necessarily enhance cooperation as the previous literature would seem to suggest [11, 36]. In the symmetric condition, agents expend the same level of effort in the inequity-aversion condition as in the selfish condition. In the asymmetric condition, inequity-aversion has a positive effect on agents’ effort expenditures when they are identical in technical capacities. Otherwise, if the agents’ capacities are heterogeneous, then the impact of inequity-aversion is correlated with the marginal effort cost (*c*); that is, it has a negative effect on their effort expenditures in projects with low marginal effort cost and a positive effect in projects with high marginal effort cost. Adding the relative kindness intention to inequity-aversion has a positive effect on the agents’ optimal effort expenditures except when *c*_4_ ≤ *c* ≤ *c*_5_ with *θ* ≠ 0.5 and *c*_6_ ≤ *c* ≤ *c*_7_ with *θ* = 0.5. Therefore, we conclude that inequity-aversion is beneficial for team cooperation only in projects where the agents have homogeneous capacities or in projects with high effort marginal cost, and this can be enhanced by adding the consideration of relative kindness intention. In contrast, in projects with low marginal effort cost, inequity-aversion is harmful to team cooperation and the consideration of relative kindness can diminish this negative impact.

Table 2 shows that, keeping the other conditions fixed and assuming inequity-aversion, the best individual effort expenditures are higher when the productivity levels are heterogeneous (***θ* ≠ 0.5**) than when they are homogeneous (***θ* = 0.5**); that is, b*|_*asym*1_ ≥ b*|_*sym*_, and $b_{i}^{*}{|}_{asym2}\geq b_{i}^{*}{|}_{asym3}$. Adding the relative kindness intentions to inequity-aversion can strengthen the positive effect of productivity heterogeneous agents on their optimal effort expenditures. These findings indicate that with inequity-aversion the agents’ difference in productivity *enhances* team cooperation. This may be because the high-productive agent’s best individual effort expenditure increases with his high marginal productivity *θ* (as shown in Table 3). Under the inequity-aversion effects, the low-productive agent would increase his effort expenditure to eliminate the negative effects of the payoff difference due to the difference in effort expenditure, which leads to the agents expending higher efforts than when they are homogeneous in their productivity. Moreover, by adding the relative kindness intention to inequity-aversion, the high-capacity agent (with high productivity) is more tolerant to the low-capacity agent (with low productivity), therefore causing him to expend a higher level of cooperative effort than the low-capacity agent. It should be noted that: In Section 3.2.2, Scenario 2, where the agents’ technical capacities are different, we assume that the productivity of the high-capacity agent (agent *i*) is not less than that of the low-capacity agent (agent *j*); that is, *θ* ≥ 0.5. Therefore, here, the high-capacity agent also represents the high-productivity agent, and the low-capacity agent represents the low-productivity agent.

From Table 3, as with the selfish condition, the effort’s marginal profit (*ηVγ*) and the agent’s marginal productivity in project output (*θ*) are positively correlated with the agents’ best individual effort expenditures. In contrast, the effort’s marginal cost (*c*) has a negative effect on the agents’ best individual expenditures. The parameters that control the agents’ degree of inequity-aversion, namely *α* and *β*, may affect the best individual effort expenditure when the agents’ capacities are different from one another. The parameter value of *β* has positive effects on the agents’ effort expenditure, whereas the value of *α* has a negative effect. In the capacity heterogeneous condition ($b_{i}^{max}\neq b_{j}^{max}$), the capacity of the low-capacity agent ($b_{j}^{max}$) has a positive effect on the effort expenditure of the high-capacity agent. By combining the results in Tables 1 and 2, we arrive at the following two conclusions. (1) In the capacity heterogeneous condition, the consideration of relative kindness intention could not only magnify the positive effect of inequity-aversion due to advantaged inequity (*β*), but could also weaken the negative effect of inequity-aversion due to disadvantaged inequity (*α*). (2) Reducing the capacity differences between the two agents enhances team cooperation and project output.

## 4. Conclusions

From the earlier models of inequity-aversion effects, we incorporated the relative kindness intention into the agent’s utility function and then investigated its effect on the agent’s effort expenditure levels in a project team. Our analyses suggest that inequity-aversion and the additional consideration of relative kindness intention provide a different account of the agents’ behavior in teamwork, especially when they are heterogeneous. We have compared the agents’ best individual effort expenditures in the selfish condition and in the inequity-aversion condition, and found the following results in the asymmetric condition: (1) inequity-aversion is beneficial for team cooperation in the case where the agents’ capacities are heterogeneous or in projects with high marginal effort cost, but is harmful to team cooperation in projects with low marginal effort cost; (2) keeping the other conditions fixed, with inequity-aversion the agents’ best individual effort expenditures show higher productivity in heterogeneous conditions than in homogeneous conditions; and (3) only in the technical capacity condition with heterogeneous agents can adding the relative kindness intention to inequity-aversion influence team cooperation by magnifying the positive effect and reducing the negative effect of inequity-aversion.

The insights from this research have potential policy implications. For example, start-up team members are generally heterogeneous in their capacities, and their team tasks are always challenging and difficult. It would seem advisable for the principal to foster inequity-aversion and the consideration of relative kindness intention in the team and let the team members know one another’s technical capacities. In contrast, in project teams with low-difficulty tasks, such as some operational projects in mature developed enterprises, it might be more beneficial for the principal to encourage team members to pursue high profits while ignoring material payoffs and relative kindness. In addition, if team members are heterogeneous in their productivity, considering the inequity caused by relative material payoff and relative kindness could facilitate intergroup cooperation.

The challenge for future research is to develop institutions and propose incentive mechanisms that effectively facilitate cooperation in teams with heterogeneous agents. Our model has not been tested empirically. This research would benefit considerably from future field studies designed to study cases in real project teams.

## On-Line appendix

Glossary of symbols are listed as following:

- *b*_*i*_: effort expended by agent *i* on the team project.
- *b*_*j*_: effort expended by agent *j* on the team project.
- *b*_*i*_′: agent *j*’s belief about the effort level that agent *i* will choose to expend.
- *b*_*j*_′: agent *i*’s belief about the effort level that agent *j* will choose to expend.
- *b*_*i*_′′: agent *i*’s belief about what agent *j* believes agent *i*’s effort choice is.
- *b*_*j*_′′: agent *j*’s belief about what agent *i* believes agent *j*’s effort choice is.
- *C*_*i*_(*b*_*i*_): agent *i*’s effort cost function, $\;C_{i}\left(b_{i}\right)=\frac{1}{2}cb_{i}{}^{2}$.
- *f*: the production function of the project, *f* = *θb*_*i*_ + (1 –*θ*)*b*_*j*_ + *γb*_*i*_ *b*_*j*_.
- *γ*: the contribution of the collaboration efforts.
- *θ*: the productivity of agent *i* on the project output.
- 1 –*θ*: the productivity of agent *j* on the project output.
- *V*: project’s value.
- *P*(*f*): the probability of the project’s success.
- *η*: the proportion of *V* that the principal shares with the agents, *η* ∈ (0,0.5].
- *Wage*: the expected monetary payoff of each agent.
- *π*_*i*_(*b*_*i*_, *b*_*j*_): the payoff of agent *i* when he expends *b*_*i*_ units of effort and agent *j* expends *b*_*j*_ units of effort.
- *ϕ*_*i*_: the kindness shown by agent *i* to agent *j*.
- $\tilde{\phi_{j}}$: agent *i*’s beliefs about the kindness that agent *j* confers on agent *i*.
- *φ*_*i*,*j*_: agent *i*’s relative kindness intention to agent *j*, $\varphi_{i,j}=\phi_{i}-\tilde{\phi_{j}}$.
- $\pi_{j}^{h}\left(b_{j}{}^{\prime }\right)$: agent *j*’s highest payoff when he expends *b*_*j*_′ units of efforts.
- $\pi_{j}^{min}\left(b_{j}{}^{\prime }\right)$: agent *j*’s lowest payoff when he expends *b*_*j*_′ units of efforts.
- $\pi_{j}^{e}\left(b_{j}{}^{\prime }\right)$: agent *j*’s equitable payoff when he expends *b*_*j*_′ units of efforts.
- $\pi_{j}^{l}\left(b_{j}{}^{\prime }\right)$: the minimum among the payoffs agent *j* can earn given *b*_*j*_′.
- Δ_*i*,*j*_: payoff difference between agents *i* and *j*, Δ_*i*,*j*_ = *π*_*i*_(*b*_*i*_, *b*_*j*_)–*π*_*j*_(*b*_*j*_, *b*_*i*_).
- *α*_*i*_: agent *i*’s inequity-averse preference which measures how much agent *i* dislikes other agents’ payoff *higher* than his (*disadvantage inequity*).
- *β*_*i*_: agent *i*’s inequity-averse preference which measures how much agent *i* dislikes other agents’ payoff *lower* than his (*advantage inequity*).

## Proofs

### Proof of Proposition 1

If the two agents choose to expend the same level of effort, denoted as *b*, then *b* should maximize their material payoffs:

$$b=\text{arg}{\text{max}}_{b}[w+\eta VP\left(f\right)-C\left(b\right)].$$

Further, we can show that:

$$b^{*}{|}_{Sa}=\{\begin{array}{rr} \frac{\eta V\tau }{2\left(c-\eta V\tau \gamma \right)}, & c>\frac{1}{2}\eta V\tau \left(1+2\gamma \right)\\ 1, & otherwise \end{array}.$$ (A-1)

If (b*|_*sa*_, b*|_*sa*_) is an equilibrium, then both agents *i* and *j* will be worse off by deviating from b*|_*sa*_. In case $c\leq \frac{1}{2}\eta V\tau \;\left(1+2\gamma \right)$ and *b* = 1, if one of the agents deviates from *b*, then he would derivate to $b-\Delta \equiv \underline{b}$. Assuming that agent *j* expends *b* units of effort, and agent *i* derivates to $\underline{b}$, agent *i*’s utility function should be given as follows:

$$U_{i}\left(\underline{b}\right)=w+\eta V\tau \left[\frac{1}{2}\underline{b}+\frac{1}{2}b+\gamma \underline{b}b\right]-\frac{1}{2}c{\underline{b}}^{2}-\frac{1}{2}c\beta \left(1+\delta \right)\left(b^{2}-{\underline{b}}^{2}\right),$$

where $\underline{b}$ should maximize agent *i*’s utility function $U_{i}\left(\underline{b}\right)$. We obtain that:

$$\underline{b}=\frac{\eta V\tau \left[1+2\gamma \right]}{2c\left[1-\beta \left(1+\delta_{i}\right)\right]},$$

where $\underline{b}$ satisfies $\underline{b}<1$.

Agent *i* would not deviate from *b* to $\underline{b}$ if

$$\Delta =U_{i}\left(b\right)-U_{i}\left(\underline{b}\right)=\eta V\tau \left(1-\underline{b}\right)\left(\frac{1}{2}+\gamma \right)-\frac{1}{2}c\left[1-\beta \left(1+\delta \right)\right]\left(1-{\underline{b}}^{2}\right)>0.$$

Substituting $\underline{b}$ into Δ, the equation above could be simplified to:

$$\begin{array}{c} \Delta U_{i}=\frac{1}{2}\left(1-\underline{b}\right)\left\{\eta V\tau \left(\frac{1}{2}+\gamma \right)-c\left[1-\beta \left(1+\delta \right)\right]\right\}\\ \overset{c\leq \frac{1}{2}\eta V\tau \;\left(1+2\gamma \right)}{\Rightarrow }>\frac{1}{2}\eta V\tau \beta \left(1+\delta \right)\left(1-\underline{b}\right)\left(\frac{1}{2}+\gamma \right)\geq 0. \end{array}$$

Since $0\leq \underline{b}<1$, *ΔU*_*i*_ is larger than or equal to 0 regardless of the value of *β*. Therefore, if $c\leq \frac{1}{2}\eta V\tau \;\left(1+2\gamma \right)$, then both agents will expend their maximal effort, that is,*b*_*i*_ = *b*_*j*_ = 1.

Similarly, we also find that in case $c>\frac{1}{2}\eta V\tau \left(1+2\gamma \right)$ and $b=\frac{\eta V\tau }{2\left(c-\eta V\tau \gamma \right)}$, if agent *i* deviates to $b-\Delta \equiv \underline{b}$, his optimal effort should be $\underline{b}=\frac{\eta V\tau \left[1+2\gamma b\right]}{2c\left[1-\beta \left(1+\delta \right)\right]}$. He will not deviate if $U_{i}\left(\underline{b}\right)<U_{i}\left(b\right)$. Let $\Delta =U_{i}\left(b\right)-U_{i}\left(\underline{b}\right)$, then

$$\Delta =\left(b-\underline{b}\right)\left\{\eta V\tau \left(\frac{1}{2}+\gamma b\right)-\frac{1}{2}c\left(b+\underline{b}\right)\left[1-\beta \left(1+\delta \right)\right]\right\}.$$

Substituting both *b* and $\underline{b}$ into Δ, we have:

$$\Delta =\frac{\eta V\tau }{4\left(c-\eta V\tau \gamma \right)}c\beta \left(1+\delta \right)\left(b-\underline{b}\right).$$

Since $b>\underline{b}$, it is easily shown that Δ > 0. So agent *i* will not deviate to $\underline{b}<b$ for ∀*β*.

Besides deviating to $\underline{b}$, in case $c>\frac{1}{2}\eta V\tau \;\left(1+2\gamma \right)$, agent *i* could deviate to $1>\bar{b}>b$. His utility if he deviates to $\bar{b}$ would be:

$$U_{i}\left(\bar{b}\right)=w+\eta V\tau \left[\frac{1}{2}\bar{b}+\frac{1}{2}b+\gamma \bar{b}b\right]-\frac{1}{2}c{\bar{b}}^{2}-\frac{1}{2}c\alpha \left(1+\delta \right)\left({\bar{b}}^{2}-b^{2}\right).$$

Assuming that $\bar{b}$ exists, we obtain: $\bar{b}=\frac{\eta V\tau \left(1+2\gamma b\right)}{4c\left[1+\alpha \left(1+\delta \right)\right]}$. Comparing agent *i*’s utility when he expends $\bar{b}$ or *b*, we find that:

$$\Delta U_{i}{}^{\text{'}}=U_{i}\left(\bar{b}\right)-U_{i}\left(b\right)=\left(\bar{b}-b\right)\left\{\eta V\tau \left(\frac{1}{2}+\gamma b\right)-\frac{1}{2}c\left(\bar{b}+b\right)\left[1+\alpha \left(1+\delta \right)\right]\right\}.$$

Let $\Delta U_{i}{}^{\prime }<0$. Then:

$$\alpha >\frac{1}{1+\delta }\left\{\frac{\eta V\tau \left(1+2\gamma b\right)}{c\left(b+\bar{b}\right)}-1\right\}.$$

Substituting *b* and $\bar{b}=b+\Delta b$ into the above equation, we further obtain that

$$\alpha >\frac{1}{1+\delta }\left\{\frac{-\eta V\tau -\Delta \text{b}\left(c-2\eta V\tau \gamma \right)}{2\eta V\tau +\Delta \text{b}\left(c-2\eta V\tau \gamma \right)}\right\}.$$

Since $c>\frac{1}{2}\eta V\tau \;\left(1+2\gamma \right)$, the right-hand side of above equation is smaller than 0. Thus, we find that $\Delta U_{i}{}^{\prime }<0$ holds for ∀*α*.

Summarizing the above analyses, we find that in the symmetric (homogenous) condition agent *i* has no incentive to deviate from *b*. The same result holds for agent *j*. Therefore, in the symmetric condition, (*b*, *b*) is an equilibrium. Similarly, we can derive the same result in case the two agents only consider inequity-aversion.

*Our claim Proved*.

### Proof of Proposition 2

Suppose that there exists an equilibrium ($b_{i}^{*}$, $b_{j}^{*}$) satisfying $b_{i}^{*}>b_{j}^{*}$. The utility functions of agent *i* and agent *j* are shown as follows:

$$U_{i}=w+\eta V\tau \left[\theta b_{i}+\left(1-\theta \right)b_{j}+\gamma b_{i}b_{j}\right]-\frac{1}{2}cb_{i}^{2}-\frac{1}{2}c\alpha \left(1+\delta \right)\left(b_{i}^{2}-b_{j}^{2}\right),$$

$$U_{j}=w+\eta V\tau \left[\theta b_{i}+\left(1-\theta \right)b_{j}+\gamma b_{i}b_{j}\right]-\frac{1}{2}cb_{j}^{2}-\frac{1}{2}c\beta \left(1+\delta \right)\left(b_{i}^{2}-b_{j}^{2}\right).$$

Maximizing the utilities of agents *i* and agent *j* yields:

$$b_{i}^{*}=\frac{\eta V\tau \left\{c\theta \left[1-\beta \left(1+\delta \right)\right]+\eta V\tau \gamma \left(1-\theta \right)\right\}}{c^{2}\left[1+\alpha \left(1+\delta \right)\right]\left[1-\beta \left(1+\delta \right)\right]-\eta^{2}V^{2}\tau^{2}\gamma^{2}},\;b_{j}^{*}=\frac{\eta V\tau \left\{c\left(1-\theta \right)\left[1+\alpha \left(1+\delta \right)\right]+\eta V\tau \gamma \theta \right\}}{c^{2}\left[1+\alpha \left(1+\delta \right)\right]\left[1-\beta \left(1+\delta \right)\right]-\eta^{2}V^{2}\tau^{2}\gamma^{2}}.$$

From $b_{i}^{*}>b_{j}^{*}$, we find that:

$$\theta >\frac{1+\alpha \left(1+\delta \right)}{2-\beta \left(1+\delta \right)+\alpha \left(1+\delta \right)},\text{ and }c>max\left\{\frac{\eta V\tau \gamma \left(2\theta -1\right)}{\left(2\theta -1\right)-\left(1+\delta \right)\left[\beta \theta +\alpha \left(1-\theta \right)\right]},\frac{\eta V\tau \gamma }{\sqrt{\left[1+\alpha \left(1+\delta \right)\right]\left[1-\beta \left(1+\delta \right)\right]}}\right\}.$$

If $\frac{1}{2}<\theta \leq \frac{1+\alpha \left(1+\delta \right)}{2-\beta \left(1+\delta \right)+\alpha \left(1+\delta \right)}$ or $c\leq \frac{\eta V\tau \gamma \left(2\theta -1\right)}{\left(2\theta -1\right)-\left(1+\delta \right)\left[\beta \theta +\alpha \left(1-\theta \right)\right]}$ or $c\leq \frac{\eta V\tau \gamma }{\sqrt{\left[1+\alpha \left(1+\delta \right)\right]\left[1-\beta \left(1+\delta \right)\right]}}$, then $b_{i}^{*}>b_{j}^{*}$ does not hold. Denote $c_{1}=\frac{\eta V\tau \gamma \left(2\theta -1\right)}{\left(2\theta -1\right)-\left(1+\delta \right)\left[\beta \theta +\alpha \left(1-\theta \right)\right]}$, $c_{2}=\frac{\eta V\tau \gamma }{\sqrt{\left[1+\alpha \left(1+\delta \right)\right]\left[1-\beta \left(1+\delta \right)\right]}}$, and $\theta_{0}=\frac{1+\alpha \left(1+\delta \right)}{2-\beta \left(1+\delta \right)+\alpha \left(1+\delta \right)}$.

Then, assuming that there exists an equilibrium $b_{i}^{*}=b_{j}^{*}=b^{*}{|}_{asym1}$, the utility functions of agent *i* and agent *j* are:

$$U_{i}=w+\eta V\tau \left[\theta b_{i}+\left(1-\theta \right)b_{j}+\gamma b_{i}b_{j}\right]-\frac{1}{2}cb_{i}^{2},$$

$$U_{j}=w+\eta V\tau \left[\theta b_{i}+\left(1-\theta \right)b_{j}+\gamma b_{i}b_{j}\right]-\frac{1}{2}cb_{j}^{2}.$$

Maximizing *U*_*i*_ and *U*_*j*_ yields:

$$b_{i}^{*}=b_{j}^{*}=b^{*}{|}_{asym1}=\{\begin{array}{ll} \frac{\eta V\tau }{c-2\eta V\tau \gamma }, & c>2\eta V\tau \gamma \\ 1, & c\leq 2\eta V\tau \gamma \end{array}.$$

Based on the above analysis, we conclude that in case *θ* > *θ*_0_ and *c* > *max*{*c*_1_, *c*_2_}, there exists two possible equilibria: the first is ($b_{i}^{*},b_{j}^{*}$) where $b_{i}^{*}>b_{j}^{*}$, and the second is (b*|_*asym*1,_ b*|_*asym*1_). Otherwise, (b*|_*asym*1,_ b*|_*asym*1_) is the possible equilibrium. Let *c*_3_ = 2*ηVτγ*, Then:

$$c_{1}-c_{3}=\frac{\eta V\tau \gamma \left\{1+2\alpha \left(1+\delta \right)-2\theta \left[1+\left(\alpha -\beta \right)\left(1+\delta \right)\right]\right\}}{\left(2\theta -1\right)-\left(1+\delta \right)\left[\beta \theta +\alpha \left(1-\theta \right)\right]}\overset{\theta <1}{\Rightarrow }>0,$$

$$c_{2}-c_{3}=\frac{\eta V\tau \gamma }{\sqrt{\left[1+\alpha \left(1+\delta \right)\right]\left[1-\beta \left(1+\delta \right)\right]}}-2\eta V\tau \gamma >\eta V\tau \gamma \left\{\frac{\alpha \left(1+\delta \right)-1}{\left[1+\alpha \left(1+\delta \right)\right]}\right\}\overset{\alpha >1}{\Rightarrow }>0.$$

Therefore, in case *c* > *max*{*c*_1_, *c*_2_}, then ($b_{i}^{*},b_{j}^{*}$) and (b*|_*asym*1,_ b*|_*asym*1_) both exist. Comparing the agents’ utility functions under ($b_{i}^{*},b_{j}^{*}$) and (b*|_*asym*1,_ b*|_*asym*1_), we find that:

$$\begin{array}{l} 2U\left(b^{*}{|}_{asym1},b^{*}{|}_{asym1}\right)-U_{i}\left(b_{i}^{*},b_{j}^{*}\right)-U_{j}\left(b_{i}^{*},b_{j}^{*}\right)\\ \;=2\eta V\tau \left\{\theta \left(b^{*}{|}_{asym1}-b_{i}\right)+\left(1-\theta \right)\left(b^{*}{|}_{asym1}-b_{j}\right)\right\}\\ \;+2\eta V\tau \left(b^{*}{|}_{asym1}{}^{2}-b_{i}b_{j}\right)\\ \;+\frac{1}{2}c\left\{b_{i}^{2}+b_{j}^{2}-2b^{*}{|}_{asym1}{}^{2}+\left(1+\delta \right)\left(\alpha +\beta \right)\left(b_{i}+b_{j}\right)\right\}o\\ \;>2\eta V\tau \left\{\theta \left(b^{*}{|}_{asym1}-b_{i}\right)+\left(1-\theta \right)\left(b^{*}{|}_{asym1}-b_{j}\right)\right\}\\ \;+2\eta V\tau \left(b^{*}{|}_{asym1}{}^{2}-b_{i}b_{j}\right)\\ \;+\frac{1}{2}c\left\{\left[1+\left(1+\delta \right)\left(\alpha +\beta \right)\right]\left(b_{i}^{2}+b_{j}^{2}\right)-2b^{*}{|}_{asym1}{}^{2}\right\}\\ \;\overset{\theta >\theta_{0},c>max\left\{c_{1},c_{2}\right\}}{\Rightarrow }>0. \end{array}$$

Thus, in case *θ* > *θ*_0_ and *c* > *max{c*_1_, *c*_2_ *}*, (b*|_*asym*1,_ b*|_*asym*1_) is an equilibrium.

*Our claim proved*.

### Proof of Corollary 1

In case *c* > 2*ηVτγ*, subtracting b*|_*asym*1_ from $b_{i}{}^{*}{|}_{S1}$ yields

$$\begin{array}{c} b^{*}{|}_{asym1}-b_{i}{}^{*}{|}_{S1}\\ \;=\frac{\eta V\tau }{\left(c-2\eta V\tau \gamma \right)\left(c^{2}-\eta^{2}V^{2}\tau^{2}\gamma^{2}\right)}\left\{\begin{array}{c} \left(1-\theta \right)\left(c^{2}-2\eta^{2}V^{2}\tau^{2}\gamma^{2}\right)\\ +\eta^{2}V^{2}\tau^{2}\gamma^{2}\theta +c\eta V\tau \gamma \left(3\theta -1\right) \end{array}\right\}\\ \;\overset{c>2\eta V\tau \gamma }{\Rightarrow }\frac{\eta V\tau }{\left(c-2\eta V\tau \gamma \right)\left(c^{2}-\eta^{2}V^{2}\tau^{2}\gamma^{2}\right)}\left\{\begin{array}{c} \left(1-\theta \right)\left(c^{2}-2\eta^{2}V^{2}\tau^{2}\gamma^{2}\right)\\ +\eta^{2}V^{2}\tau^{2}\gamma^{2}\left(7\theta -2\right) \end{array}\right\}>0. \end{array}$$

We find that $b^{*}{|}_{asym1}>b_{i}{}^{*}{|}_{S1}$. Since $b_{i}{}^{*}{|}_{S1}>b_{j}{}^{*}{|}_{S1}$, then $b^{*}>b_{i}{}^{*}{|}_{S1}>b_{j}{}^{*}{|}_{S1}$. In case *ηVτγ* < *c* ≤ 2*ηVτγ*, then b* = 1, and $b^{*}{|}_{asym1}>b_{i}{}^{*}{|}_{S1}>b_{j}{}^{*}{|}_{S1}$; if *c* ≤ *ηVτγ*, then $b^{*}=b_{i}{}^{*}{|}_{S1}=b_{j}{}^{*}{|}_{S1}=1$.

*Our claim proved*.

### Proof of Corollary 2

Taking the derivate of b* with respect to *ηV*, *γ* and *c*, we obtain that:

$$\frac{\partial b^{*}}{\partial \eta V}=\frac{c}{c-2\eta V\tau \gamma },\;\frac{\partial b^{*}}{\partial \gamma }=\frac{2\eta^{2}V^{2}\tau^{2}}{{\left(c-2\eta V\tau \gamma \right)}^{2}},\;\frac{\partial b^{*}}{\partial c}=-\frac{\eta V\tau }{{\left(c-2\eta V\tau \gamma \right)}^{2}}.$$

Since *c* > 2*ηVτγ*, we obtain: $\frac{\partial b^{*}}{\partial \eta V\tau }>0,\;\frac{\partial b^{*}}{\partial \gamma }>0,\;\frac{\partial b^{*}}{\partial c}<0.$

*Our claim proved*.

### Proof of Proposition 3

Assume that there exists an optimal effort level $\bar{b_{i}^{*}}$ that satisfies $\bar{b_{i}^{*}}>b_{j}^{max}$ and maximizes agent *i*’s utility. According to our pervious assumptions, the utility function of agent *i* could be written as follows:

$$U_{i}=\pi_{i}-\frac{1}{2}c\alpha \left(1-\delta \right)\left(b_{i}^{*}{}^{2}-b_{j}^{max}{}^{2}\right).$$

Maximizing agent *i*’s utility *U*_*i*_, we obtain:

$$b_{i}^{*}=\bar{b_{i}^{*}}=\frac{\eta V\tau \left(\theta +\gamma b_{j}^{max}\right)}{c\left[1+\alpha \left(1-\delta \right)\right]}.$$

From $\bar{b_{i}^{*}}>b_{j}^{max}$, we can further get that $c<\frac{\eta V\tau \left(\theta +\gamma b_{j}^{max}\right)}{\left[1+\alpha \left(1-\delta \right)\right]b_{j}^{max}}$.

Denote $c_{4}=\frac{\eta V\tau \left(\theta +\gamma b_{j}^{max}\right)}{\left[1+\alpha \left(1-\delta \right)\right]b_{j}^{max}}$. Assume that *c* < *c*_4_. Then, comparing agent *i*’s utility in case he expends $\bar{b_{i}^{*}}$ and in case he expends $b_{j}^{max}$, we have:

$$U_{i}\left(\bar{b_{i}^{*}},b_{j}^{max}\right)-U_{i}\left(b_{j}^{max},b_{j}^{max}\right)=\frac{1}{2}\left(\bar{b_{i}^{*}}-b_{j}^{max}\right)\left\{\eta V\tau \left(\theta +\gamma b_{j}^{max}\right)-cb_{j}^{max}\left[1+\alpha \left(1-\delta \right)\right]\right\}.$$

If *c* < *c*_4_, then $U_{i}\left(\bar{b_{i}^{*}},b_{j}^{max}\right)>U_{i}\left(b_{j}^{max},b_{j}^{max}\right)$, and $b_{i}^{*}=\bar{b_{i}^{*}}$ is the optimal effort level of agent *i*. Otherwise, if *c* ≥ *c*_4_, then we can show that $U_{i}\left(\bar{b_{i}^{*}},b_{j}^{max}\right)\leq U_{i}\left(b_{j}^{max},b_{j}^{max}\right)$, and $b_{i}^{*}=b_{j}^{max}$ is agent *i*’s optimal effort expenditure.

Similarly, assume that there exists an optimal effort level $\underline{b_{i}^{*}}$ satisfying $\underline{b_{i}^{*}}<b_{j}^{max}$. Then, the utility function of agent *i* could be written as:

$$U_{i}=\pi_{i}-\frac{1}{2}c\beta \left(1+\delta \right)\left(b_{j}^{max}{}^{2}-b_{i}^{*}{}^{2}\right).$$

Maximizing *U*_*i*_ yields:

$$b_{i}^{*}=\underline{b_{i}^{*}}=\frac{\eta V\tau \left(\theta +\gamma b_{j}^{max}\right)}{c\left[1-\beta \left(1+\delta \right)\right]}.$$

From $\underline{b_{i}^{*}}<b_{j}^{max}$, we find that: $c>\frac{\eta V\tau \left(\theta +\gamma b_{j}^{max}\right)}{\left[1-\beta \left(1+\delta \right)\right]b_{j}^{max}}$. Denote $c_{5}=\frac{\eta V\tau \left(\theta +\gamma b_{j}^{max}\right)}{\left[1-\beta \left(1+\delta \right)\right]b_{j}^{max}}$. Assume *c* > *c*_5_. Comparing agent *i*’s utility in case he expends $\underline{b_{i}^{*}}$ and in case he expends $b_{j}^{max}$, we obtain:

$$U_{i}\left(\underline{b_{i}^{*}},b_{j}^{max}\right)-U_{i}\left(b_{j}^{max},b_{j}^{max}\right)=\frac{1}{2}\left(b_{j}^{max}-\underline{b_{i}^{*}}\right)\left\{cb_{j}^{max}\left[1-\beta \left(1+\delta \right)\right]-\eta V\tau \left(\theta +\gamma b_{j}^{max}\right)\right\}.$$

From the above equation, we find that if *c* > *c*_5_, then $U_{i}\left(\underline{b_{i}^{*}},b_{j}^{max}\right)>U_{i}\left(b_{j}^{max},b_{j}^{max}\right)$, that is $\underline{b_{i}^{*}}$ is the optimal effort choice of agent *i*.

Moreover, from the above analysis we also find that there does not exist a situation that satisfies both $\bar{b_{i}^{*}}>b_{j}^{max}$ and $\underline{b_{i}^{*}}<b_{j}^{max}$. Therefore, in case *c*_4_ ≤ *c* ≤ *c*_5_, then $b_{j}^{max}$ is agent *i*’s optimal effort expenditure.

*Our claim proved*.

### Proof of Proposition 4

As the proof in this case is quite similar to the proof of Proposition 3, it is omitted.

### Proof of Corollary 3

From $\bar{b_{i}^{*}}$ and $\underline{b_{i}^{*}}$, we find that

$$\frac{\partial \bar{{\text{b}}_{\text{i}}^{*}}}{\partial \delta }=\frac{\alpha \eta V\tau \left(\theta +\gamma b_{j}^{max}\right)}{c{\left[1+\alpha \left(1-\delta \right)\right]}^{2}}>0\text{ and }\frac{\partial \underline{{\text{b}}_{\text{i}}^{*}}}{\partial \delta }=\frac{\beta \eta V\tau \left(\theta +\gamma b_{j}^{max}\right)}{c{\left[1-\beta \left(1-\delta \right)\right]}^{2}}>0.$$

*Our claim proved*.

### Proof of Corollary 4

According to the agents’ utility functions, agent *i*’s optimal effort level without inequity-aversion is:

$$b_{i}^{*}{}^{Sb}=\frac{1}{c}\eta V\tau \left(\theta +\gamma b_{j}^{max}\right).$$

Comparing $b_{i}^{*}{}^{Sb}$ with $\bar{b_{i}^{*}}$ and $\underline{b_{i}^{*}}$, we find that $b_{i}^{*}{}^{Sb}>\bar{b_{i}^{*}}$ and $b_{i}^{*}{}^{Sb}<\underline{b_{i}^{*}}$. Therefore, in case *c* < *c*_1_, then $b_{i}^{*}{}^{Sb}>\bar{b_{i}^{*}}$, so that inequity-aversion and consideration of relative kindness intention reduce agent *i*’s optimal effort. In the case that *c* > *c*_2_ and $b_{i}^{*}{}^{Sb}<\underline{b_{i}^{*}}$, so that inequity-aversion and consideration of relative kindness intention could increase agent *i*’s optimal effort; otherwise, $b_{i}^{*}=b_{j}^{max}$.

Comparing $b_{j}^{max}$ and $b_{i}^{*}{}^{Sb}$, we obtain that:

$$b_{i}^{*}{}^{Sb}-b_{j}^{max}=\frac{1}{c}\eta V\tau \left(\theta +\gamma b_{j}^{max}\right)-b_{j}^{max}.$$

Define $c_{8}=\frac{1}{b_{j}^{max}}\eta V\tau \left(\theta +\gamma b_{j}^{max}\right)$.

We find that:

1. in case *c* < *c*_8,_ then $b_{i}^{*}{}^{Sb}>b_{j}^{max}$, so that inequity-aversion and consideration of relative kindness intention decrease agent *i*’s optimal effort;
2. in case *c* > *c*_8_, then $b_{i}^{*}{}^{Sb}<b_{j}^{max}$, so that inequity-aversion and consideration of relative kindness intention increase agent *i*’s optimal effort;
3. in case *c = c*_8_, then $b_{i}^{*}{}^{Sb}=b_{j}^{max}$, so that inequity-aversion and consideration of relative kindness intention have no effect on agent *i*’s effort expenditure.

Combining with Corollary 3, Corollary 4 is proved.

*Our claim proved*.

### Proof of Corollary 5

In addition *δ*, the project’s profit share and characteristics, such as *η*, *V*, *γ*, *θ*, *c*, and $b_{j}^{max}$ may also affect agent’s optimal effort choice. Taking derivatives yields:

$$\frac{\partial \bar{b_{i}^{*}}}{\partial \eta }>0,\;\frac{\partial \underline{b_{i}^{*}}}{\partial \eta }>0,\;\frac{\partial \bar{b_{i}^{*}}}{\partial V}>0,\;\frac{\partial \underline{b_{i}^{*}}}{\partial V}>0,\;\frac{\partial \bar{b_{i}^{*}}}{\partial \gamma }>0,\;\frac{\partial \underline{b_{i}^{*}}}{\partial \gamma }>0,\;\frac{\partial \bar{b_{i}^{*}}}{\partial \theta }>0,\;\frac{\partial \underline{b_{i}^{*}}}{\partial \theta }>0,\;\frac{\partial \bar{b_{i}^{*}}}{\partial c}<0,\;\frac{\partial \underline{b_{i}^{*}}}{\partial c}<0,\;\frac{\partial \bar{b_{i}^{*}}}{\partial b_{j}^{max}}>0,\;\frac{\partial \underline{b_{i}^{*}}}{\partial b_{j}^{max}}>0.$$

*Our claim proved*.
